# Synthetic approaches to multifunctional indenes

**DOI:** 10.3762/bjoc.7.204

**Published:** 2011-12-29

**Authors:** Neus Mesquida, Sara López-Pérez, Immaculada Dinarès, Ermitas Alcalde

**Affiliations:** 1 Laboratori de Química Orgànica, Departament de Farmacologia i Química Terapèutica, Facultat de Farmàcia, Universitat de Barcelona, Avda. Joan XXIII s/n, 08028 Barcelona, Spain

**Keywords:** aldol reaction, amides, indanones, indenes, organometallic reagents

## Abstract

The synthesis of multifunctional indenes with at least two different functional groups has not yet been extensively explored. Among the plausible synthetic routes to 3,5-disubstituted indenes bearing two different functional groups, such as the [3-(aminoethyl)inden-5-yl)]amines, a reasonable pathway involves the (5-nitro-3-indenyl)acetamides as key intermediates. Although several multistep synthetic approaches can be applied to obtain these advanced intermediates, we describe herein their preparation by an aldol-type reaction between 5-nitroindan-1-ones and the lithium salt of *N*,*N*-disubstituted acetamides, followed immediately by dehydration with acid. This classical condensation process, which is neither simple nor trivial despite its apparent directness, permits an efficient entry to a variety of indene-based molecular modules, which could be adapted to a range of functionalized indanones.

## Introduction

Compounds with an indene core are of great interest as precursors of metallocene complexes for catalytic polymerization processes, as well as being present in *N*-heterocyclic carbene ligands and functional materials [1–9]. In addition, indene-based structures are a source of bioactive compounds in drug discovery and development [10–18]. The routes to access multiply substituted indenes with at least two different functional groups (FGs) are generally complex, and the synthetic approaches leading to these compounds lag behind those for heteroaromatic systems, e.g., indoles, being limited as it is by the scarce knowledge of indene chemistry in comparison with heterocyclic chemistry [3,17–22]. Moreover, chemical transformation by indenes, which could permit rapid access to different substitution patterns, is rather cumbersome owing to the complexity of the indene chemistry, especially of those processes that could be modulated by the aromatic character of a resulting indenyl anion species. Indeed, indene is an unusually acidic nonaromatic carbocyclic system and, although structurally different, indene and indole show similar p*K*_a_ values of 20.1 and 21.0 in dimethylsulfoxide solution, respectively [23–24].

**Scheme 1:**
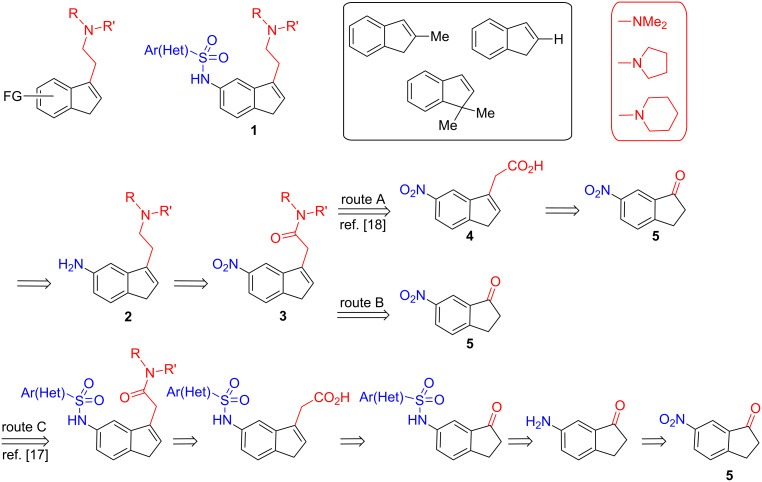
Retrosynthetic pathways to 3,5-disubstituted indenes bearing two functional groups: Indenylsulfonamides **1**.

During the course of our studies on indene-based ligands of general type **1** with biological effects on the central nervous system, we found that different stepwise synthetic routes could be applied to inden-5-amines **2** bearing a disubstituted *N*,*N*-aminoethyl side arm at the indene 3-position [17–18]. After analyzing reasonable synthetic routes to the [3-(aminoethyl)inden-5-yl)]amine intermediates **2** starting from substituted indan-1-one **5**, route A was chosen as the most suitable way of functionalizing the 5-position of the indene, e.g., by changing the aryl(heteroaryl) structural motif of a sulfonamide moiety (Scheme 1). This route proceeded in three steps, and allowed the representative (3-indenyl)acetic acids **4** to be conveniently prepared from nitroindanones **5** and the lithium salt of ethyl acetate [18].

The present work deals with the alternative synthetic route B, which relies on the direct formation of the advanced (3-indenyl)acetamides **3** from nitroindanone **5** and shortens the synthetic sequence to two steps. The aldol-type reaction of indanone **5** with the lithium salts of different *N*,*N*-disubstituted acetamides, followed immediately by dehydration afforded the (3-indenyl)acetamides **3**. The success of this route, however, depends on two main factors: (i) The nature of the lithiated base used to deprotonate acetamides in order to form lithium enolates, which needs to be considered when adjusting the reaction conditions [25]; and (ii) the chemical response of indanoles towards dehydration [6,18] to give the desired *endo*-olefin **3**, which should be favored with the presence of a methyl group at the indene 2-position.

## Results and Discussion

We recently reported that the reaction of 2-methylindanone **6** with the lithium salt of ethyl acetate (generated in situ by the reaction of lithium hexamethyldisilazide with ethyl acetate), immediately followed by dehydration and hydrolysis/isomerization, afforded the acetic acid **7** with an improved yield of 74%. Compound **7** was then conveniently transformed to the corresponding acetamide **8**. The reduction of the amide group with AlH_3_·NMe_2_Et was the critical step for the preparation of the key inden-5-amine **9** due to the particularly troublesome isolation process [18].

**Scheme 2:**
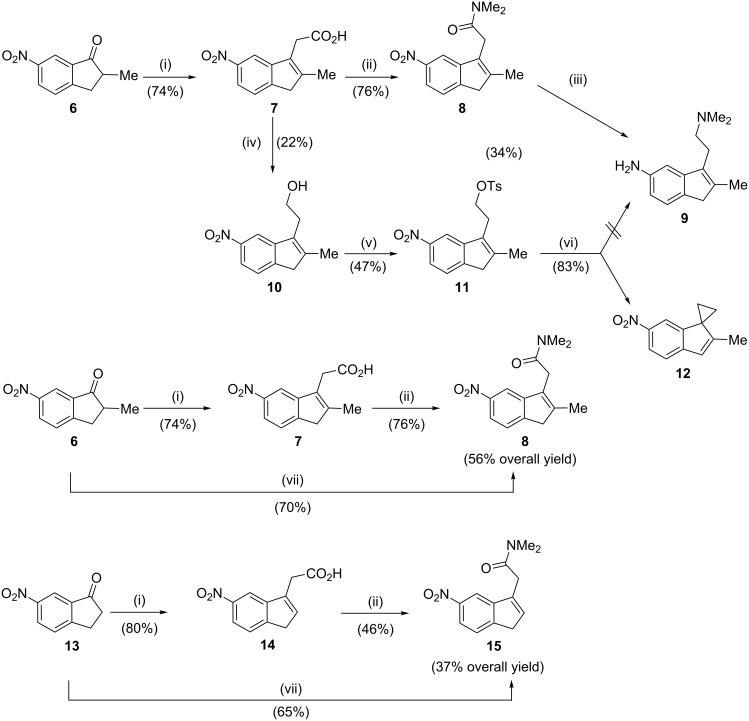
Reagents and conditions: (i) (a) EtOAc, LHMDS, THF, −78 °C, (b) H_2_SO_4_, H_2_O, 60 °C [18]; (ii) (a) SOCl_2_, CH_2_Cl_2_, reflux, (b) Me_2_NH, rt [18]; (iii) (a) AlH_3_·NMe_2_Et, THF, 0 °C, (b) Zn, AcOH, rt [18]; (iv) AlH_3_·NMe_2_Et, THF, 0 °C; (v) TsCl, Py, CH_2_Cl_2_, rt; (vi) Me_2_NH, DMF, rt; (vii) (a) *N,N*-dimethylacetamide, LDA, THF, −78 °C, (b) TFA, CH_2_Cl_2_, rt, 2 h.

Thus, a different synthetic option was examined in which the (3-indenyl)acetic acid **7** was reduced to the corresponding alcohol **10** and transformed to tosylate **11**. However, an attempt to convert the (3-indenyl)ethylsulfonate **11** into the advanced inden-5-amine **9** was ineffective, resulting in the formation of the spiro indene **12** instead, and hence this route was not studied further (Scheme 2 and Supporting Information File 1). It should be mention that the propensity of several 3-substituted indenes, appropriately fitted with leaving groups, to undergo spirocyclization has been previously reported [26–27].

To shorten the multistep route A, an alternative preparation of the acetamide **8** was examined, based on an aldol-type reaction between nitroindanone **6** and the lithium salt of *N*,*N*-dimethylacetamide. After various reaction conditions and lithiated bases were examined, the condensation of **6** with the α-lithio-*N*,*N*-dimethylacetamide (prepared in turn from *N*,*N*-dimethylacetamide and an excess of lithium diisopropylamide), immediately followed by dehydration with trifluoroacetic acid, afforded indenylacetamide **8** in 70% yield, which was 14% higher than the previously reported result obtained with the (3-indenyl)acetic acid **7** [18]. Upon application of the same experimental conditions to indanone **13**, the yield of indenylacetamide **15** was improved from 37% [18] to 65% (Scheme 2 and Supporting Information File 1).

**Scheme 3:**
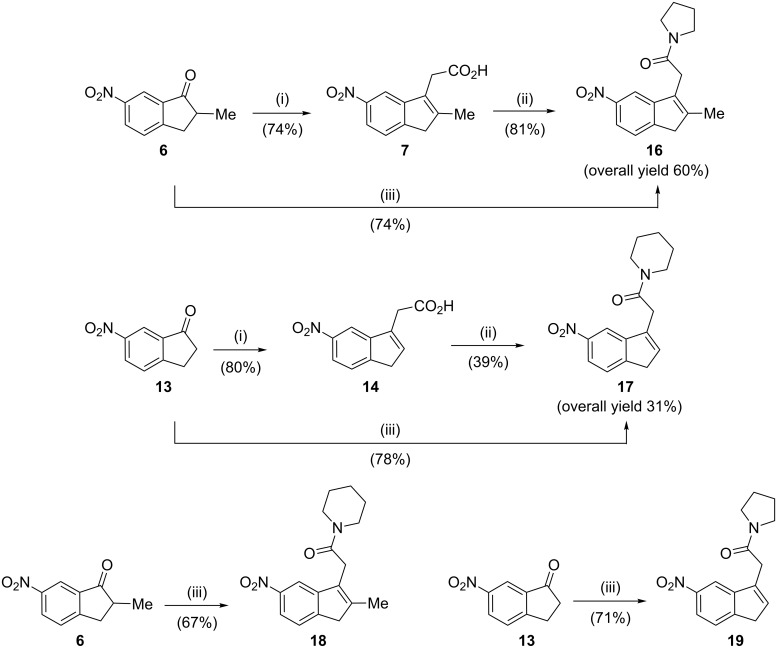
Reagents and conditions: (i) (a) EtOAc, LHMDS, THF, −78 °C, (b) H_2_SO_4_, H_2_O, 60 °C [18]; (ii) (a) SOCl_2_, CH_2_Cl_2_, reflux, (b) pyrrolidine or piperidine, rt [7]; (iii) (a) *N*-acetylpyrrolidine or *N*-acetylpiperidine, LDA, −78 °C; (b) TFA, CH_2_Cl_2_, rt, 17 h.

We next studied the utility of this aldol-type reaction protocol by using other disubstituted acetamides, such as *N*-acetylpyrrolidine and *N*-acetylpiperidine, as shown in Scheme 3. When the reaction conditions used to prepare *N*,*N*-dimethyl-(3-indenyl)acetamide **8** were applied, 2-methylindanone **6** failed to give the (3-indenyl)acetamide **16**, and did not progress beyond the corresponding indanol (Supporting Information File 1). When the dehydration process was prolonged to 17 h at room temperature, conversion of indanone **6** to the desired acetamide **16** proceeded in 74% yield, which was 14% higher than that previously reported for a two-step procedure with the (3-indenyl)acetic acid **7** [18]. Following this modified procedure, the reaction between indanones **6** or **13** and the lithium salt of *N*-acetylpiperidine or *N*-acetylpyrrolidine afforded indenylacetamides **17**–**19** in good yield (≥67%).

**Scheme 4:**
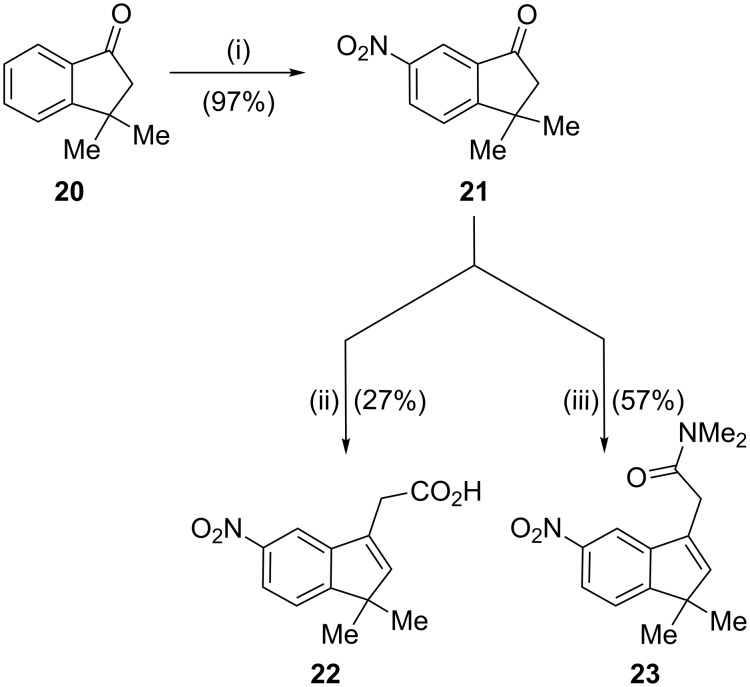
Reagents and conditions: (i) KNO_3_, H_2_SO_4_, −5 °C; (ii) (a) EtOAc, LHMDS, THF, −78 °C, (b) H_2_SO_4_, H_2_O, 60 °C, 7 h [18]; (iii) (a) *N*,*N*-dimethylacetamide, LDA, THF, −78 °C, (b) TFA, CH_2_Cl_2_, rt, 17 h.

Finally, to broaden the scope of this aldol-type reaction, we examined the synthesis of (1,1-dimethyl-3-indenyl)acetamides. Following a standard nitration protocol applied to different indanones [17], the 3,3-dimethylindanone **20** was efficiently transformed into the corresponding 6-nitroindan-1-one **21** in 97% yield and no traces of the isomer 4-nitroindanone were detected, probably due to the presence of the dimethyl moiety at the indanone 3-position in **20**. This was a marked improvement on a previously reported 62% yield of nitroindanone **21** employing a different nitration method [28]. In our recently reported preliminary experiments, the conversion of **21** to the corresponding (3-indenyl)acetic acid **22** resulted in a fairly low yield of 27%, and the transformation to *N*,*N*-dimethyl-(3-indenyl)acetamide **23** was not carried out [18]. However, after the synthetic approach was changed from route A to route B, the indenylacetamide **23** was prepared by aldol-type condensation between indanone **21** and the lithium salt of *N*,*N*-dimethylacetamide, in 57% yield (Scheme 4).

We thus successfully optimized the synthesis of (3-indenyl)acetamides **3**, obtaining higher yields with a shorter procedure.

The crude reaction products of the aldol-type reaction between nitroindanones and α-lithioacetamides often contained minor amounts of *exo*-olefins. Pure indenylacetamides were isolated after careful column chromatography, and sometimes a high-throughput flash purification system was necessary.

**Figure 1:**
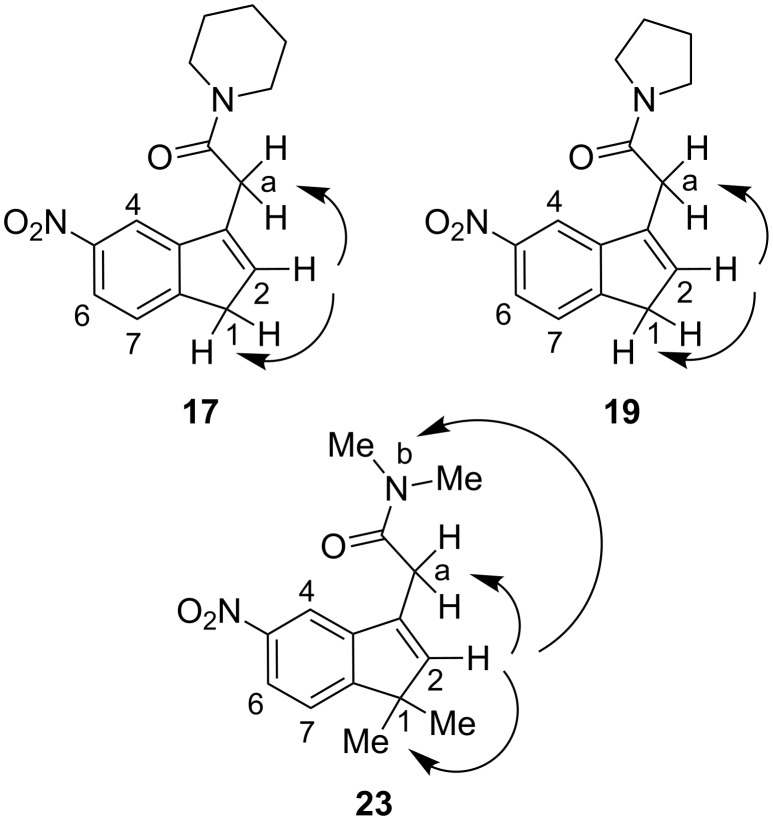
Key NMR responses for compounds **17**, **19** and **23**: 1D NOESY experiments.

The structures of the new compounds were confirmed by spectroscopic methods, and their ^1^H NMR and ^13^C NMR chemical shifts and physical data are gathered in the experimental description (Supporting Information File 1). The constitution of the acetamides **17**, **19** and **23** was determined by 1D NOESY experiments in CDCl_3_ at 500 MHz. Thus, for each compound, irradiation at the H-2 proton of the indene core revealed a NOE for the methylene protons H-1 and H-a. For compound **23**, an additional NOE was observed at the methyl protons of the acetamide group (Figure 1).

## Conclusion

Among different synthetic routes that could lead to disubstituted indenes **1** bearing two functional groups, such as the indenamines **2**, a feasible path explored here required the use of (5-nitro-3-indenyl)acetamides **3**, prepared either from (5-nitro-3-indenyl)acetic acid (**4**) or 5-nitroindan-1-one (**5**). The best option to prepare the advanced indenylacetamides **3** was based on an aldol-type reaction between nitroindanone **5** and the lithium salt of *N*,*N*-disubstituted acetamides, followed immediately by dehydration with acid. Although apparently direct, the classical aldol-type condensation process is neither simple nor rudimentary when applied to members of the indene family. Particularly significant aspects of the reported transformation are (i) the nature of the lithiated base used to form the lithium enolate, and (ii) the dehydration process to obtain the desired *endo*-olefin **3**. The optimal experimental results were achieved with lithium diisopropylamide as the base and by employing a long dehydration reaction time, which provided the targeted indenylacetamides **3** in good yield. This synthetic protocol can be exploited for the elaboration of a variety of indene-based molecular modules with applications in fields as diverse as bioactive compounds, ligand precursors for metallocene catalyst systems, and functional materials.

## Supporting Information

File 1 Assays related to the preparation of **8**, **10**, **11** and **16**, experimental details, characterization data and copies of NMR and ESI-HRMS spectra of all new compounds.

## References

[1] Chirik P J (2010). Organometallics.

[2] Zhang C, Luo F, Cheng B, Li B, Song H, Xu S, Wang B (2009). Dalton Trans.

[3] Guo S, Liu Y (2008). Org Biomol Chem.

[4] Guan Z-H, Ren Z-H, Zhao L-B, Liang Y-M (2008). Org Biomol Chem.

[5] Enders M, Baker R W (2006). Curr Org Chem.

[6] Silver S, Leppänen A-S, Sjöholm R, Penninkangas A, Leino R (2005). Eur J Org Chem.

[7] Leino R, Lehmus P, Lehtonen A (2004). Eur J Inorg Chem.

[8] Zargarian D (2002). Coord Chem Rev.

[9] Hummel S, Kirsch S F (2011). Beilstein J Org Chem.

[10] Waldmann H, Karaguni I-M, Carpintero M, Gourzoulidou E, Herrmann C, Brockmann C, Oschkinat H, Müller O (2004). Angew Chem, Int Ed.

[11] Karaguni I-M, Glüsenkamp K-H, Langerak A, Geisen C, Ullrich V, Winde G, Möröy T, Müller O (2002). Bioorg Med Chem Lett.

[12] Li B-F, Moree W J, Yu J, Coon T, Zamani-Kord S, Malany S, Jalali K, Wen J, Wang H, Yang C (2010). Bioorg Med Chem Lett.

[13] Moree W J, Li B-F, Jovic F, Coon T, Yu J, Gross R S, Tucci F, Marinkovic D, Zamani-Kord S, Malany S (2009). J Med Chem.

[14] Hodgson D M, Winning L H (2008). Beilstein J Org Chem.

[15] Huffman J W, Padgett L W (2005). Curr Med Chem.

[16] Böhme T M, Keim C, Kreutzmann K, Linder M, Dingermann T, Dannhardt G, Mutschler E, Lambrecht G (2003). J Med Chem.

[17] Alcalde E, Mesquida N, Frigola J, López-Pérez S, Mercè R (2008). Org Biomol Chem.

[18] Alcalde E, Mesquida N, López-Pérez S, Frigola J, Mercè R (2009). J Med Chem.

[19] Wang J, Zhang L, Jing Y, Huang W, Zhou X (2009). Tetrahedron Lett.

[20] Tsukamoto H, Ueno T, Kondo Y (2007). Org Lett.

[21] Ivchenko P V, Nifant’ev I E, Luzikov Yu N, Mkoyan S G (2007). Synthesis.

[22] Nishikata T, Kobayashi Y, Kobayshi K, Yamamoto Y, Miyaura N (2007). Synlett.

[23] Bordwell F G, Drucker G E, Fried H E (1981). J Org Chem.

[24] Bordwell F G, Fried H E (1991). J Org Chem.

[25] Rathman T L, Bailey W F (2009). Org Process Res Dev.

[26] Kelly P A, Berger G O, Wyatt J K, Nantz M H (2003). J Org Chem.

[27] Resconi L, Piemontesi F, Camurati I, Balboni D, Sironi A, Moret M, Rychlicki H, Zeigler R (1996). Organometallics.

[28] Koelsch C F, LeClaire C D (1941). J Org Chem.

