# Supplementary material for: Synthetic approaches to multifunctional indenes
Source: Beilstein J Org Chem. 2011 Dec 29;7:1739–44. doi: 10.3762/bjoc.7.204 (PMC3252879; doi:10.3762/bjoc.7.204)

Supporting Information
for
Synthetic approaches to multifunctional indenes

Neus Mesquida*, Sara López-Pérez, Immaculada Dinarès and Ermitas Alcalde*

Address: Laboratori de Química Orgànica, Departament de Farmacologia i Química Terapèutica, Facultat de Farmàcia, Universitat de Barcelona, Avda. Joan XXIII s/n, 08028 Barcelona, Spain

Email: Neus Mesquida - neusmesquida@ub.edu; Ermitas Alcalde - ealcalde@ub.edu

* Corresponding author

Assays related to the preparation of 8, 10, 11 and 16, experimental details, characterization data and copies of NMR and ESI-HRMS spectra of all new compounds

Table of contents

- Assays related to the preparation of alcohol **10**……………………………..…..…......S2
- Assays related to the preparation of tosylate **11**……………………………..…..…......S2
- Assays related to the preparation of acetamide **8**…………………………....…..…......S2
- Assays related to the preparation of acetamide **16**…………………………..…..…......S3
- Experimental section…………………………………………………..………......S4
- NMR spectra and ESI-HRMS spectra of new compounds……………………….......S9

Assays related to the preparation of alcohol 10

- **2-(2-Methyl-5-nitro-1*H*-inden-3-yl)ethanol (10)**

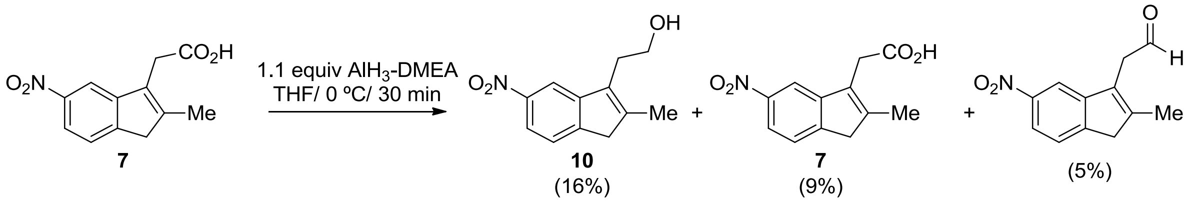

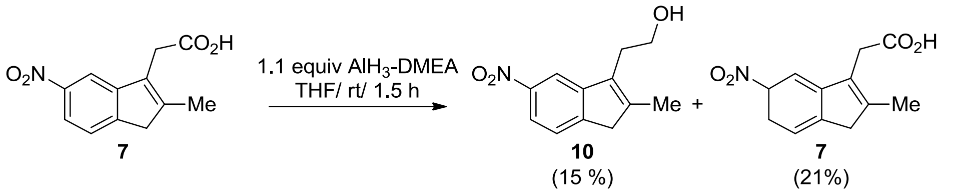

Assays related to the preparation of tosylate 11

- **2-(2-Methyl-5-nitro-1*H*-inden-3-yl)ethyl 4-methylbenzenesulfonate (11)**

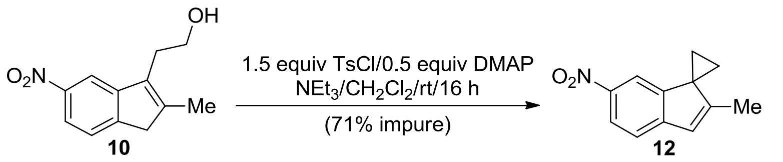

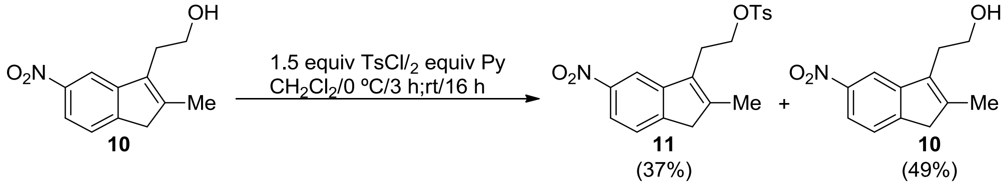

Assays related to the preparation of acetamide 8

- ***N,N*-Dimethyl-2-(2-methyl-5-nitro-1*H*-inden-3-yl)acetamide (8)**

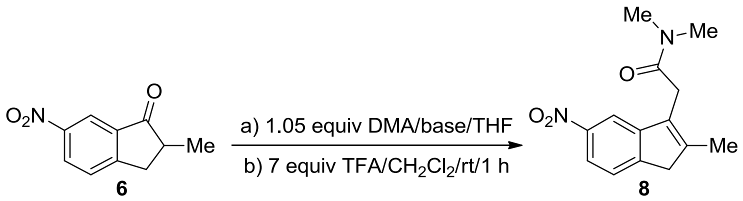

assay[a]	base (equiv)	T (°C)	time (h)	rt[b] (%)
1	2.1 equiv LHMDS	−78	1.5	62%
2	2.1 equiv LHMDS	0	3	35%
3	1.1 equiv LDA	−78	3	50%
4	1.1 equiv LDA	0	3	–
5	2.1 equiv LDA	−78	2.5	70%

[a]1 equiv of 2-methyl-6-nitroindan-1-one (**6**).

[b]Yield isolated after purification by chromatography.

Assays related to the preparation of acetamide 16

- **2-Methyl-6-nitro-1-(2-oxo-2-pyrrolidin-1-ylethyl)indan-1-ol**

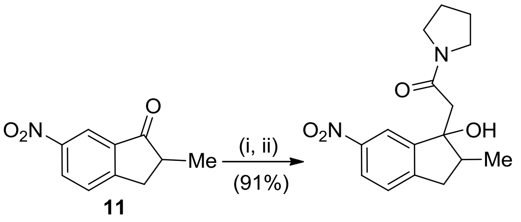

Reagents and conditions: (i) *N*-acetylpyrrolidine, LDA, −78 °C, (ii) TFA, CH_2Cl_2, rt, 2 h.

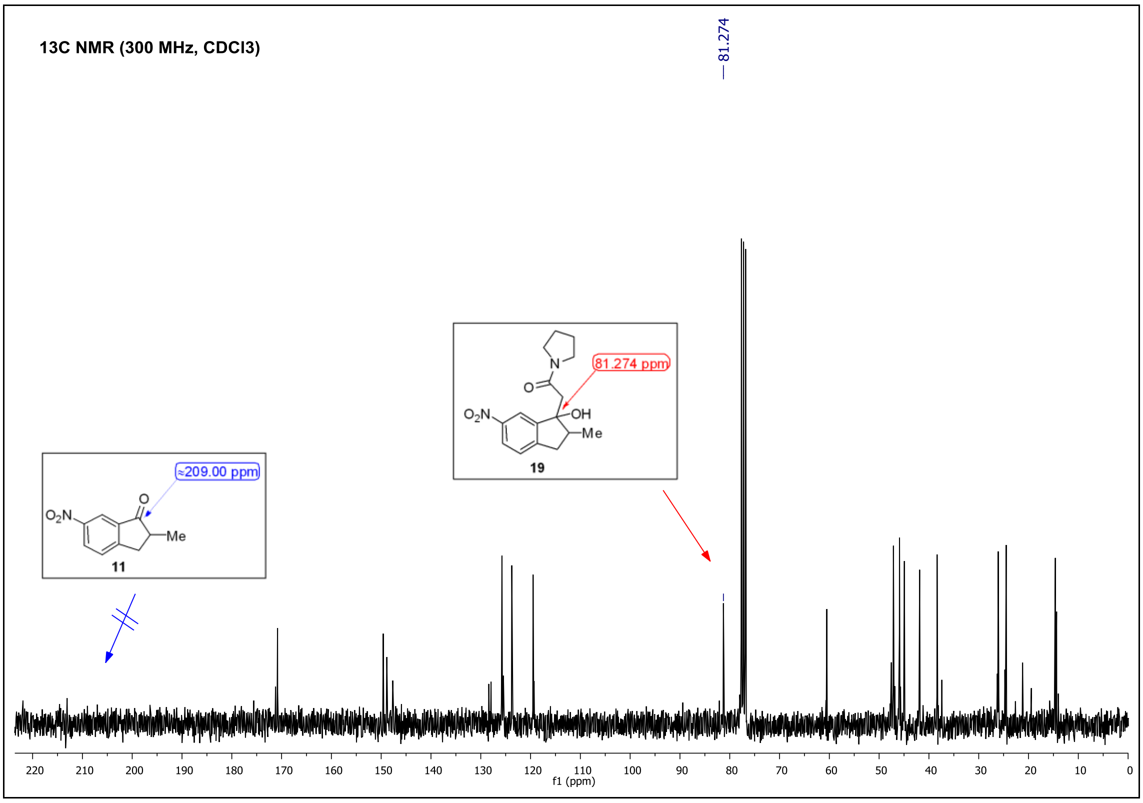

Experimental section

1. General information

Reaction yields are not optimized. All reagents obtained from commercial sources were used without purification. Melting point: Gallenkamp Melting Point Apparatus MPD350.BM2.5 with digital thermometer/uncorrected. IR (KBr disk or thin film): Nicolet 320 FT spectrophotometer. ^{1}H NMR: Varian Gemini 300 (300 MHz), Mercury 400 (400 MHz) and Inova 500 (500 MHz) spectrometers at 298 K. Chemical shifts were referenced and expressed in ppm (δ) relative to the central peak of TMS for chloroform-*d*. ^{13}C NMR: Varian Gemini 300 (75.4 MHz) and Mercury 400 (100.6 MHz) spectrometers at 298 K. Chemical shifts were referenced and expressed in ppm (δ) relative to the central peak of chloroform-d (77.0 ppm). MS were obtained by using EI at 70 eV in a Hewlett-Packard spectrometer (HP-5989A model). Microanalyses were performed on a Carlo Erba 1106 analyzer. ESI-HRMS: Mass spectra were obtained on an Agilent LC/MSD-TOF spectrometer. TLC: Merck precoated silica gel 60 F254 plates with UV light (254 nm) as a visualizing agent and/or H_2PtCl_2 3% aq./KI 10% aq. (1:1) or $KMnO_4$ ethanolic solution. Column chromatography was performed on silica gel 60 ACC 35–70 μm Chromagel (SDS). VersaFlash™ (Sigma-Aldrich) was used as a high-throughput flash purification system.

2. Materials

3,3-Dimethyl-2,3-dihydro-1*H*-inden-1-one (**20**), *N*,*N*-dimethylacetamide, *N*-acetylpyrrolidine, *N*-acetylpiperidine are commercially available. 2-Methyl-6-nitroindan-1-one (**6**) [18], 2-(2-methyl-5-nitro-1*H*-inden-3-yl)acetic acid (**7**) [1], 3-[2-(dimethylamino)ethyl]-2-methyl-1*H*-inden-5-amine (**9**) [1], 6-nitroindan-1-one (**13**) [1], 2-(5-nitro-1*H*-inden-3-yl)acetic acid (**14**) [1], and 2-(1,1-dimethyl-5-nitro-1*H*-inden-3-yl)acetic acid (**22**) [1] were prepared as previously described.

3. 2-(2-Methyl-5-nitro-1*H*-inden-3-yl)ethanol (10)

To dry THF (30 mL) cooled to 0 °C, AlH_3–NMe_2Et (0.5 M in toluene, 7.10 mL, 3.54 mmol) was added. Then, a solution of acid **7** (0.75 g, 3.21 mmol) in dry THF (30 mL) cooled to 0 °C was added. At the end of the addition, the mixture was maintained at the same temperature under argon atmosphere for 2 h. The reaction was quenched with THF:H_2O (1:1, 75 mL) and the temperature was allowed to rise slowly to room temperature. The resulting suspension was filtered through Celite®. The layers were separated and the organic extract was washed with brine (3 × 50 mL), dried over anhydrous Na_2SO_4, filtered and evaporated to dryness. The residue obtained was purified by silica gel column chromatography (CH_2Cl_2:MeOH mixtures of increasing polarity as eluent). Alcohol **10** was obtained as a yellow solid (154.0 mg, 22%).

Mp 120−121 °C; IR (KBr disk): ν (OH) 3245; ν (NO_2) 1511, 1337 cm^{-1}; ^{1}H NMR (300 MHz, $CDCl_3$): δ 2.16 (s, 3H), 2.86 (t, *J* = 6.6 Hz, 1H), 3.41 (s, 2H), 3.85 (t, *J* = 6.6 Hz, 2H), 7.45 (d, *J* = 6.0 Hz, 1H), 8.02 (dd, *J* = 2.1, 8.4 Hz, 1H), 8.06 (d, *J* = 2.1 Hz, 1H) ppm; ^{13}C NMR ($CDCl_3$, 100.6 MHz): δ 14.3 (CH_3), 28.7 (CH_2), 42.8 (CH_2), 61.2 (CH_2), 112.9 (CH), 119.4 (CH), 123.3 (CH), 133.0, 144.5, 147.5, 147.8, 149.5 ppm; EI-MS *m/z* (%): 219 (94) [$M^{+\bullet}$], 201 (87) [$M^{+\bullet}$ – 18], 141 (100) [$M^{+\bullet}$ – 78], 115 (64) [$M^{+\bullet}$ – 104].

4. 2-(2-Methyl-5-nitro-1*H*-inden-3-yl)ethyl 4-methylbenzenesulfonate (11)

To a stirred suspension of the alcohol **10** (0.10 g, 0.46 mmol) in dry CH_2Cl_2 (1 mL) cooled to 0 °C, a solution of pyridine (0.14 mL, 1.82 mmol) and 4-methylbenzenesulfonyl chloride (0.19 g, 1.0 mmol) was added under argon atmosphere, and the resulting mixture was stirred at room temperature for 24 h. The reaction mixture was diluted with CH_2Cl_2 (10 mL) and washed with water (3 × 5 mL). The organic extract was dried with anhydrous Na_2SO_4, filtered and evaporated to dryness. The residue obtained was purified by silica gel column chromatography (CH_2Cl_2:MeOH mixtures of increasing polarity as eluent). Tosylate derivate **11** was obtained as an orange solid (80.0 mg, 47%).

Mp 109−110 °C; IR (KBr disk): ν (NO_2) 1517, 1340; ν (O-SO_2-) 1356, 1172 cm^{-1}; ^{1}H NMR (300 MHz, $CDCl_3$): δ 2.08 (s, 3H), 2.36 (s, 3H), 2.89 (t, *J* = 6.9 Hz, 2H), 3.31 (s, 2H), 4.19 (t, *J* = 6.9 Hz, 2H), 7.19 (dd, *J* = 0.8, 8.0 Hz, 2H), 7.40 (d, *J* = 8.1 Hz, 1H), 7.63 (d, *J* = 8.1 Hz, 1H), 7.77 (d, *J* = 2.1 Hz, 1H), 7.95−7.99 (m, 1H) ppm; ^{13}C NMR ($CDCl_3$, 75.4 MHz): δ 14.2 (CH_3), 21.5 (CH_3), 25.0 (CH_2), 42.7 (CH_2), 67.9 (CH_2), 112.3 (CH), 119.3 (CH), 123.3 (CH), 127.6 (CH), 129.6 (CH), 130.1, 132.6, 144.7, 145.7, 146.9, 147.3, 149.3 ppm; EI-MS *m/z* (%): 373 (9) [$M^{+\bullet}$], 201 (100) [$M^{+\bullet}$ – 172].

5. 2'-Methyl-6'-nitrospiro[cyclopropane-1,1'-indene] (12)

To a stirred solution of compound **11** (80.0 mg, 0.21 mmol) in dry DMF (1.5 mL) was added a solution of dimethylamine (40% in water, 0.30 mL, 2.14 mmol) under argon atmosphere and the resulting mixture was stirred at room temperature for 21 h. Then, the mixture was concentrated in vacuum. The residue was dissolved with EtOAc (20 mL) and washed with water (3 × 15 mL). The organic extract was dried with anhydrous Na_2SO_4, filtered and evaporated to dryness. Spiroindene **12** was obtained as a yellow solid (35.0 mg, 83%).

Mp 49−50 °C; IR (KBr disk): ν (NO_2) 1512, 1326 cm^{-1}; ^{1}H NMR (400 MHz, $CDCl_3$): δ 1.60−1.63 (m, 2H), 1.71−1.74 (m, 2H), 1.90 (s, 3H), 6.68 (s, 1H), 7.36 (d, *J* = 6.3 Hz, 1H), 7.78 (s, 1H), 8.12 (dd, *J* = 0.8, 8.0 Hz, 1H) ppm; ^{13}C NMR ($CDCl_3$, 100.6 MHz): δ 12.3 (CH_2), 15.3 (CH_3), 35.0, 112.8 (CH), 119.8 (CH), 121.9 (CH), 124.1 (CH), 148.7, 150.0, 155.3 ppm; EI-MS *m/z* (%): 201

(100) [$M^{+\bullet}$], 153 (48) [$M^{+\bullet}$ – 48], 128 (53) [$M^{+\bullet}$ – 73]. ESI(+)-HRMS calc. for $C_{12}H_{11}NO_2$ $[M + H]^+$: 202.0862; found: 202.0863.

6. 3,3-Dimethyl-6-nitroindan-1-one (21)

3,3-Dimethylindan-1-one (**20**) (5.0 g, 31.21 mmol) was added in one portion to 95–97% H_2SO_4 (10 mL) at 0 °C. A solution of KNO_3 (3.47 g, 34.33 mmol) in 95–97% H_2SO_4 (30 mL) was added dropwise. The mixture was stirred for 1 h at –5 °C and then poured over 500 mL of ice. The mixture was stirred at room temperature for 2 h. The resultant solid was filtered to give 3,3-dimethyl-6-nitroindan-1-one (**21**) (6.24 g, 97%) as a beige solid.

Mp 102−103 °C; IR (KBr disk): ν(C=O) 1717; ν(NO_2) 1530, 1352 cm^{-1}; ^1H NMR (300 MHz, $CDCl_3$): δ 1.50 (s, 6H), 2.72 (s, 2H), 7.70 (dd, *J* = 0.7, 9.0 Hz, 1H), 8.46–8.50 (m, 2H) ppm; ^{13}C NMR ($CDCl_3$, 75.4 MHz): δ 29.8 (CH_3), 39.3, 53.1 (CH_2), 118.9 (CH), 124.9 (CH), 129.3 (CH), 136.4, 148.0, 169.2, 203.4 (C=O) ppm. EI-MS *m/z* (%): 205 (37) [$M^{+\bullet}$], 190 (100) [$M^{+\bullet}$ – 15], 115 (31) [$M^{+\bullet}$ – 90].

7. Synthesis of 2-(5-nitro-1H-inden-3-yl)acetamides. General procedure

To a sufficient amount of dry THF cooled to −78 °C, a solution of lithium diisopropylamide (LDA, 2.1 equiv) was added, under argon atmosphere. Then, *N,N*-dimethylacetamide, *N*-acetylpyrrolidine or *N*-acetylpiperidine (1.05 equiv) was added and the resulting mixture was stirred at −78 °C for 1 h. Finally, a solution of 2-methyl-6-nitroindan-1-one (**6**), 6-nitroindan-1-one (**13**) or 3,3-dimethyl-6-nitroindan-1-one (**21**) (1 equiv) in the sufficient amount of dry THF was added, and the resulting mixture was kept at –78 °C for 2 h. The reaction mixture was acidified with 1N HCl and extracted with EtOAc. The organic extracts were dried with anhydrous Na_2SO_4, filtered and evaporated to dryness. To a stirred solution of the previous residue in dry CH_2Cl_2, cooled to 0 °C, was added trifluoroacetic acid (7.0 equiv) and the resulting mixture was stirred for 2 or 17 h. The reaction mixture was evaporated to dryness. The residue obtained was basified with saturated $NaHCO_3$ aqueous solution and extracted with CH_2Cl_2. The organic extract, after being dried over anhydrous Na_2SO_4 and filtered, was evaporated to dryness. The residue obtained was purified by silica gel column chromatography (CH_2Cl_2:EtOAc mixtures of increasing polarity as eluent).

N,N-Dimethyl-2-(2-methyl-5-nitro-1*H*-inden-3-yl)acetamide (8)

The above procedure was followed with *N,N*-dimethylacetamide (0.25 mL, 2.74 mmol), LDA (1.5 M in THF, 3.66 mL, 5.49 mmol), 2-methyl-6-nitroindan-1-one (**6**) (0.50 g, 2.61 mmol) in dry THF (30 mL) and TFA (1.70 mL) in dry CH_2Cl_2 (25 mL) for 2 h. Acetamide derivative **8** (475 mg,

70%) was obtained as a yellow solid. The spectral data of **8** were identical to those previously reported [1].

_N,N_-Dimethyl-2-(5-nitro-1_H_-inden-3-yl)acetamide (15)

The procedure described above was followed with _N,N_-dimethylacetamide (0.26 mL, 2.96 mmol), LDA (1.5 M in THF, 3.95 mL, 5.93 mmol), 6-nitroindan-1-one (**13**) (0.50 g, 2.82 mmol) in dry THF (30 mL) and TFA (1.51 mL) in dry CH_2Cl_2 (25 mL) for 2 h. Acetamide derivative **15** (451 mg, 65%) was obtained as a yellow solid. The spectral data of **15** were identical to those previously reported [1].

2-(2-Methyl-5-nitro-1_H_-inden-3-yl)-1-(pyrrolidin-1-yl)ethanone (16)

The above procedure was followed with _N_-acetylpyrrolidine (0.30 mL, 2.75 mmol), LDA (1.5 M in THF, 3.66 mL, 5.49 mmol), 2-methyl-6-nitroindan-1-one (**6**) (0.50 g, 2.61 mmol) in dry THF (30 mL) and TFA (1.81 mL) in dry CH_2Cl_2 (30 mL) for 17 h. Acetamide derivative **16** (550 mg, 74%) was obtained as a yellow solid. The spectral data of **16** were identical to those previously reported [1].

2-(5-Nitro-1_H_-inden-3-yl)-1-(piperidin-1-yl)ethanone (17)

The above procedure was followed with _N_-acetylpiperidine (0.38 mL, 2.96 mmol), LDA (1.5 M in THF, 3.95 mL, 5.93 mmol), 6-nitroindan-1-one (**13**) (0.50 g, 2.82 mmol) in dry THF (30 mL) and TFA (1.81 mL) in dry CH_2Cl_2 (25 m) for 17 h. Acetamide derivative **17** (627 mg, 78%) was obtained as a brown solid. The spectral data of **17** were identical to those previously reported [1].

2-(2-Methyl-5-nitro-1_H_-inden-3-yl)-1-(piperidin-1-yl)ethanone (18)

The above procedure was followed with _N_-acetylpiperidine (0.35 mL, 2.75 mmol), LDA (1.5 M in THF, 3.66 mL, 5.49 mmol), 2-methyl-6-nitroindan-1-one (**6**) (0.50 g, 2.61 mmol) in dry THF (30 mL) and TFA (1.61 mL) in dry CH_2Cl_2 (30 mL) for 17 h. Acetamide derivative **18** (535 mg, 67%) was obtained as a yellow solid.

Mp 89−90 °C; IR (KBr disk): ν (N−C=O) 1642; ν (NO_2) 1519, 1342 cm^{-1}; ^{1}H NMR (300 MHz, $CDCl_3$): δ 1.56−1.59 (m, 4H), 1.64−1.69 (m, 2H), 2.13 (s, 3H), 3.44 (s, 2H), 3.51−3.54 (m, 2H), 3.57−3.61 (m, 4H), 7.44 (d, J = 8.1 Hz, 1H), 7.99−8.04 (m, 2H) ppm; ^{13}C NMR ($CDCl_3$, 75.4 MHz): δ 14.4 (CH_3), 24.4 (CH_2), 25.5 (CH_2), 26.4 (CH_2), 30.4 (CH_2), 42.9 (CH_2), 43.0 (CH_2), 47.0 (CH_2), 113.4 (CH), 119.4 (CH), 123.1 (CH), 130.7, 144.3, 147.5, 147.8, 149.1, 167.6 (C=O) ppm. ESI(+)-HRMS calc. for $C_{17}H_{21}N_2O_3$ [M + H]$^+$: 301.1547; found: 301.1543.

2-(5-Nitro-1*H*-inden-3-yl)-1-(pyrrolidin-1-yl)ethanone (19)

The above procedure was followed with *N*-acetylpyrrolidine (0.33 mL, 2.96 mmol), LDA (1.5 M in THF, 3.95 mL, 5.93 mmol), 6-nitroindan-1-one (**13**) (0.50 g, 2.82 mmol) in dry THF (30 mL) and TFA (1.69 mL) in dry CH_2Cl_2 (25 mL) for 17 h. Acetamide derivative **19** (543 mg, 71%) was obtained as a yellow solid.

Mp 118−119 °C. IR (KBr disk): ν(N−C=O) 1641; ν(NO_2) 1515, 1340 cm^{-1}. ^1H NMR (500 MHz, $CDCl_3$): δ 1.89−1.92 (m, 2H), 1.96−2.02 (m, 2H), 3.48 (d, *J* = 1.0 Hz, 2H), 3.51−3.54 (m, 2H), 3.67 (d, *J* = 1.5 Hz, 2H), 6.55 (t, *J* = 1.5 Hz, 1H), 7.54 (d, *J* = 8.0 Hz, 1H), 8.09 (dd, *J* = 2.2, 8.2 Hz, 1H), 8.19 (d, *J* = 2.0 Hz, 1H) ppm. ^{13}C NMR ($CDCl_3$, 75.4 MHz): δ 24.4 (CH_2), 26.2 (CH_2), 34.4 (CH_2), 38.2 (CH_2), 45.9 (CH_2), 47.0 (CH_2), 114.4 (CH), 120.3 (CH), 123.9 (CH), 133.8 (CH), 137.3, 142.3, 146.2, 147.4, 151.0, 167.9 (C=O), 167.9 (C=O) ppm. ESI(+)-HRMS calc. for $C_{15}H_{17}N_2O_3$ [M + H]$^+$: 273.1234; found: 273.1233.

2-(1,1-Dimethyl-5-nitro-1*H*-inden-3-yl)-*N,N*-dimethylacetamide (23)

The above procedure was followed with *N,N*-dimethylacetamide (0.71 mL, 7.67 mmol), LDA (1.5 M in THF, 10.2 mL, 15.35 mmol), 3,3-dimethyl-6-nitroindan-1-one (**21**) (1.5 g, 7.31 mmol) in dry THF (90 mL) and TFA (3.98 mL) in dry CH_2Cl_2 (50 mL) for 17 h. Acetamide derivative **23** (1.13 g, 56%) was isolated as a yellow solid.

Mp 71−72 °C; IR (KBr disk): ν(N−C=O) 1643; ν(NO_2) 1522, 1341 cm^{-1}; ^1H NMR (500 MHz, $CDCl_3$): δ 1.34 (s, 6H), 3.00 (s, 3H), 3.06 (s, 3H), 3.58 (s, 2H), 6.31 (s, 1H), 7.40 (d, *J* = 8.5 Hz, 1H), 8.07 (d, *J* = 2.0 Hz, 1H), 8.10 (dd, *J* = 2.0, 7.5 Hz, 1H) ppm; ^{13}C NMR ($CDCl_3$, 75.4 MHz): δ 24.1 (CH_3), 33.1 (CH_3), 35.6 (CH_2), 37.7 (CH), 48.9, 114.7 (CH), 121.1 (CH), 121.4 (CH), 132.9, 144.1, 146.2 (CH), 147.6, 160.5, 169.5 (C=O) ppm. ESI(+)-HRMS calc. for $C_{15}H_{19}N_2O_3$ [M + H]$^+$: 275.1390; found: 275.1388.

References

1. Alcalde, E.; Mesquida, N.; López-Pérez, S.; Frigola, J.; Mercè, R. *J. Med. Chem.* **2009,** *52,* 675–687. doi:10.1021/jm8009469

NMR and ESI-HRMS spectra of new compounds

- **2'-Methyl-6'-nitrospiro[cyclopropane-1,1'-indene] (12)**

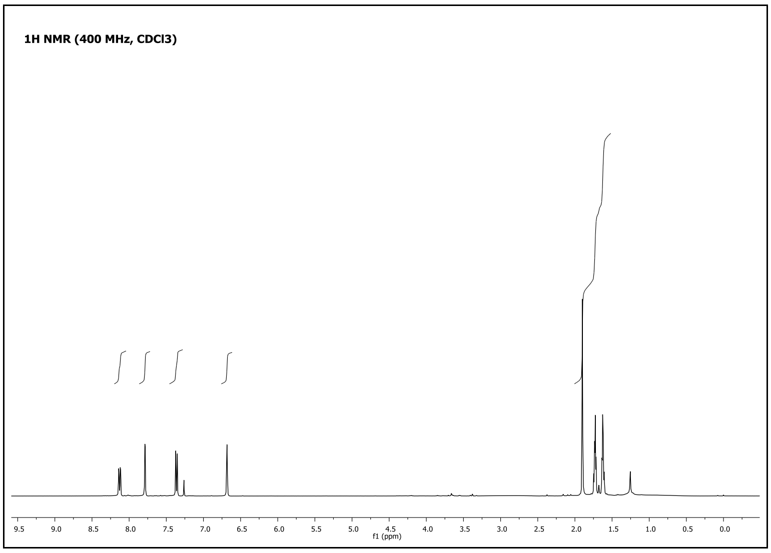

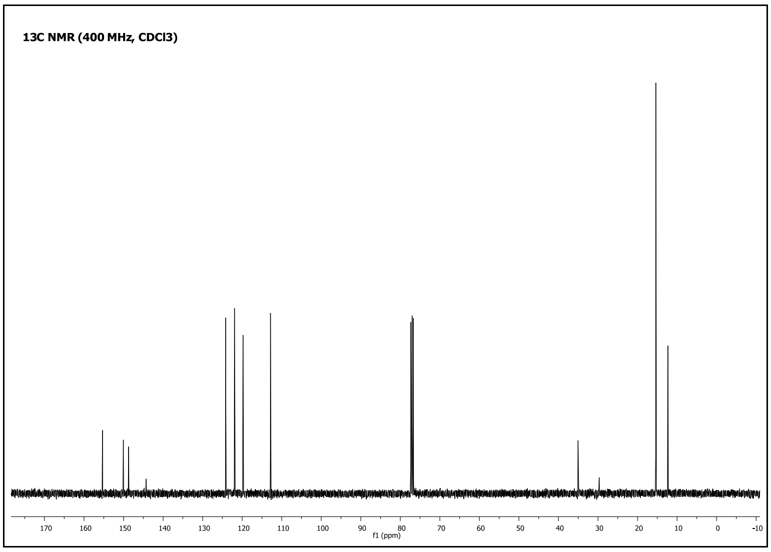

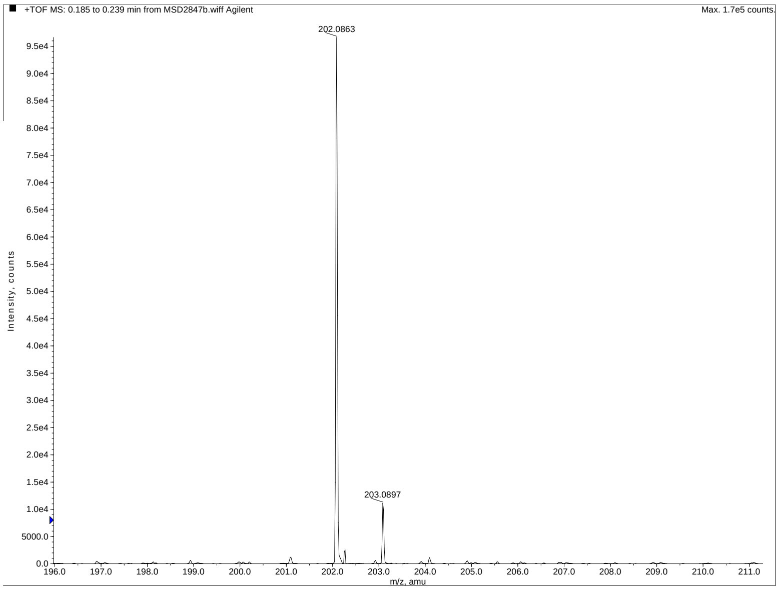

- **2-(5-Nitro-1*H*-inden-3-yl)-1-(piperidin-1-yl)ethanone (17)**

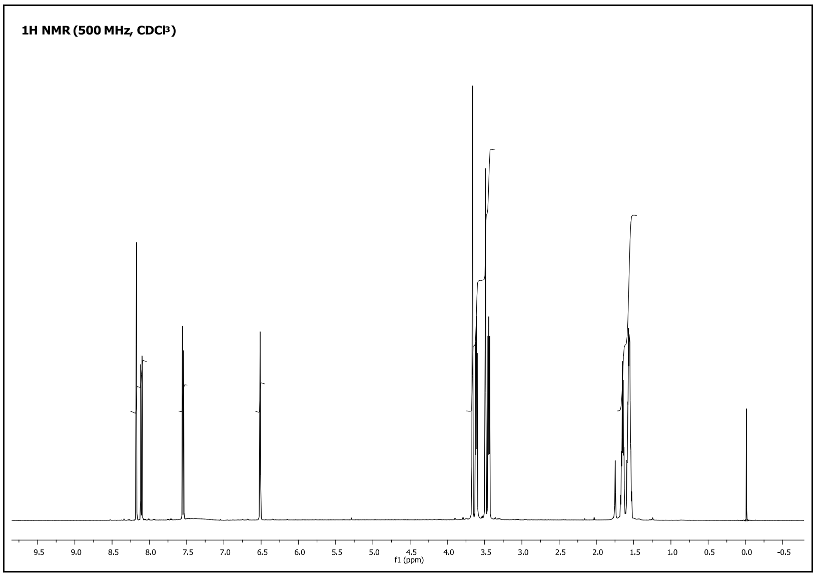

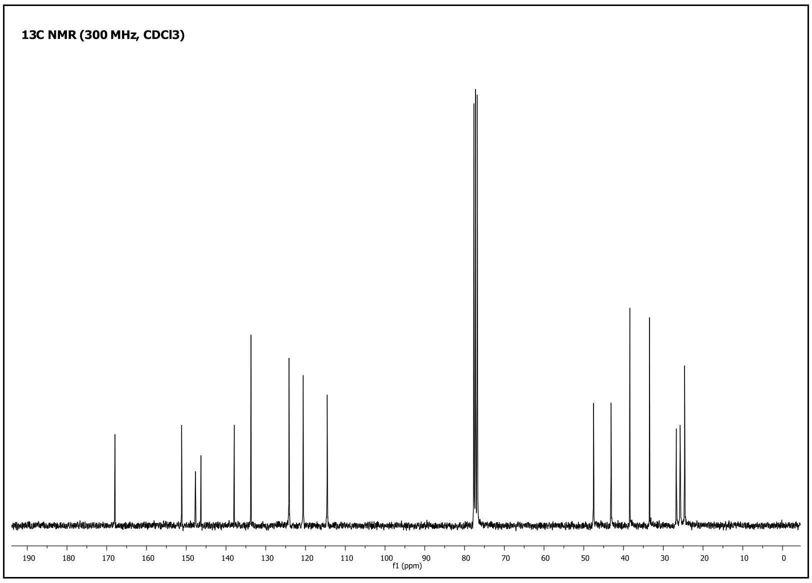

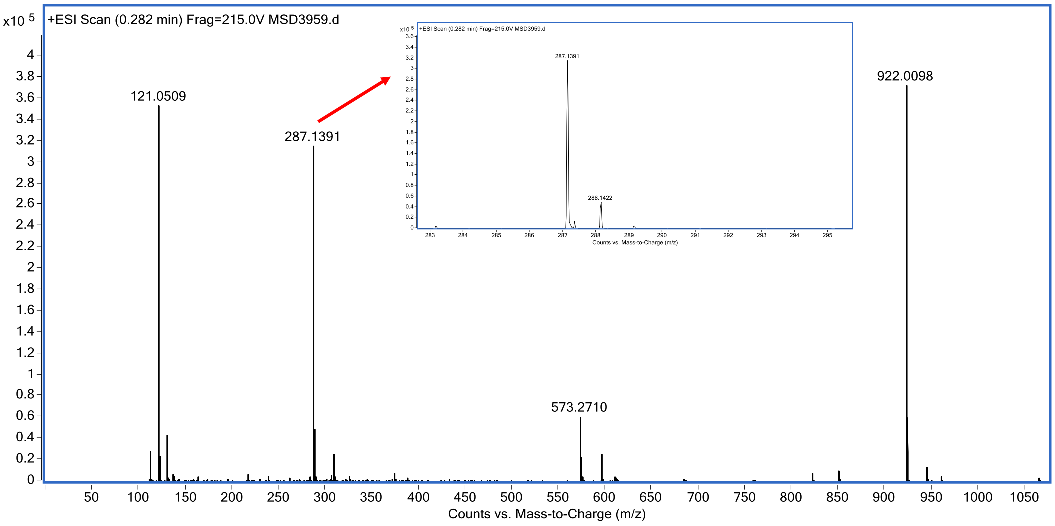

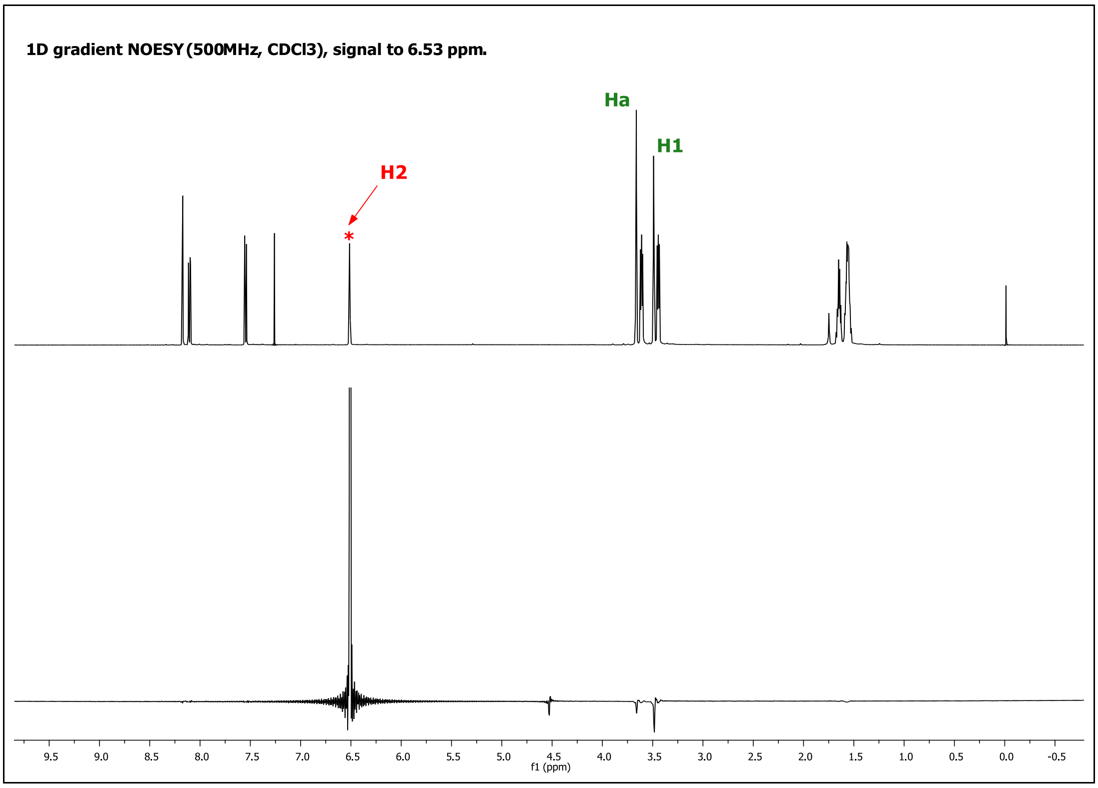

1D gradient NOESY (500MHz, CDCl3), signal to 6.53 ppm.
Ha
H1
H2
*
9.5
9.0
8.5
8.0
7.5
7.0
6.5
6.0
5.5
5.0
4.5
4.0
3.5
3.0
2.5
2.0
1.5
1.0
0.5
0.0
-0.5
f1 (ppm)

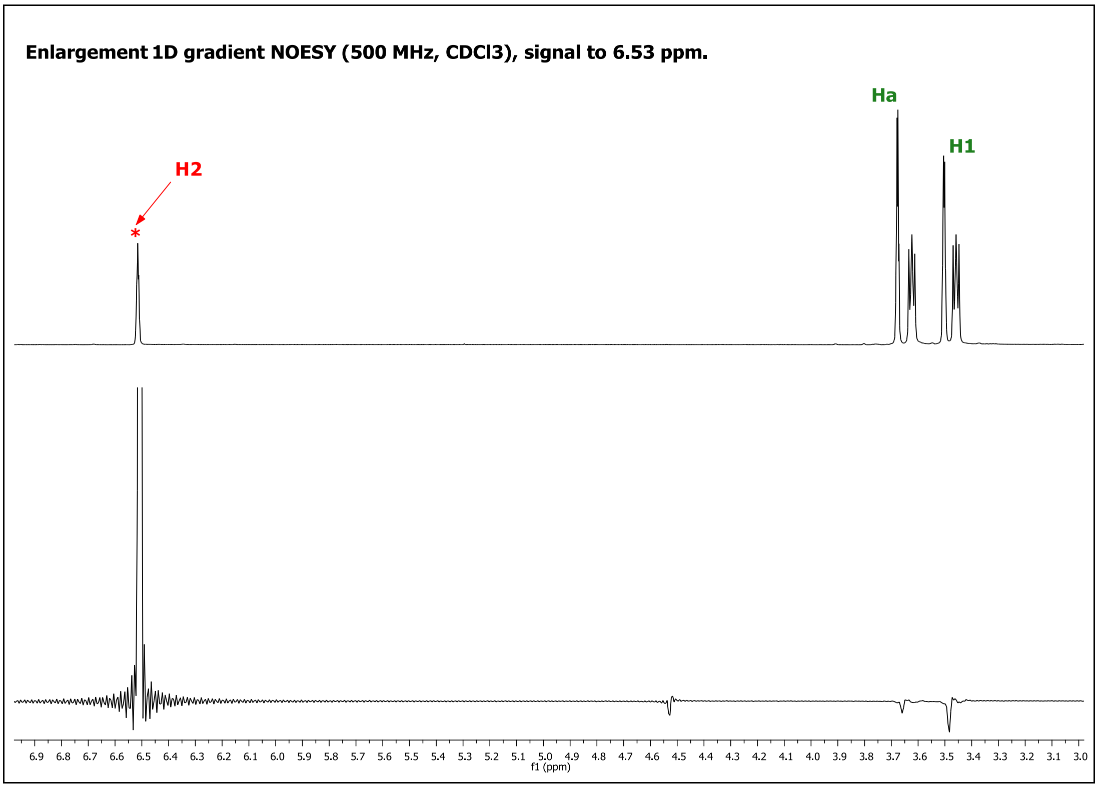

Enlargement 1D gradient NOESY (500 MHz, CDCl3), signal to 6.53 ppm.
Ha
H1
H2
*
6.9 6.8 6.7 6.6 6.5 6.4 6.3 6.2 6.1 6.0 5.9 5.8 5.7 5.6 5.5 5.4 5.3 5.2 5.1 5.0 4.9 4.8 4.7 4.6 4.5 4.4 4.3 4.2 4.1 4.0 3.9 3.8 3.7 3.6 3.5 3.4 3.3 3.2 3.1 3.0
f1 (ppm)

- **2-(2-Methyl-5-nitro-1*H*-inden-3-yl)-1-(piperidin-1-yl)ethanone (18)**

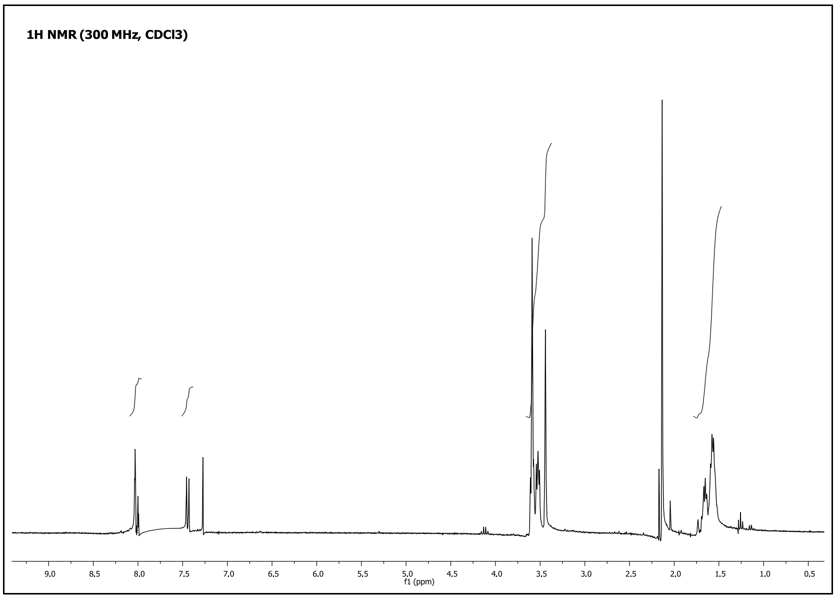

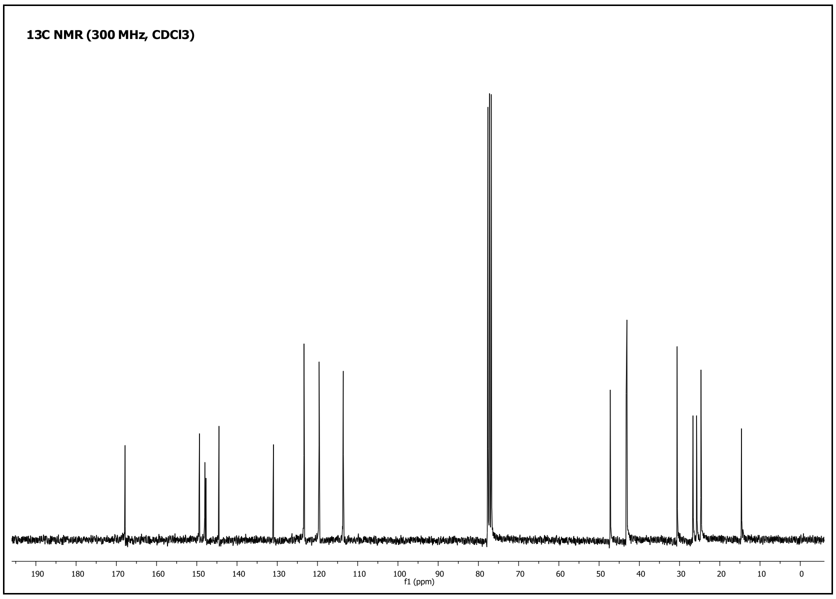

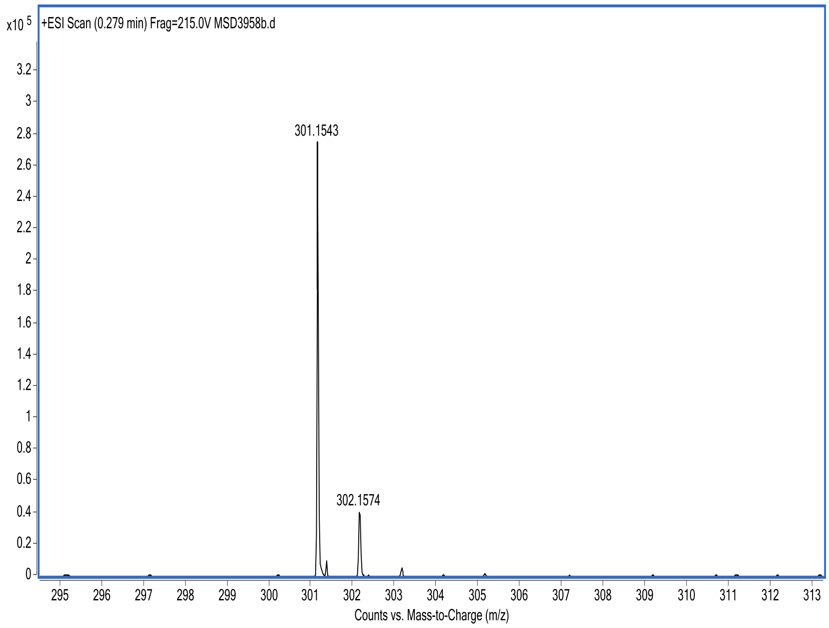

- **2-(5-Nitro-1*H*-inden-3-yl)-1-(pyrrolidin-1-yl)ethanone (19)**

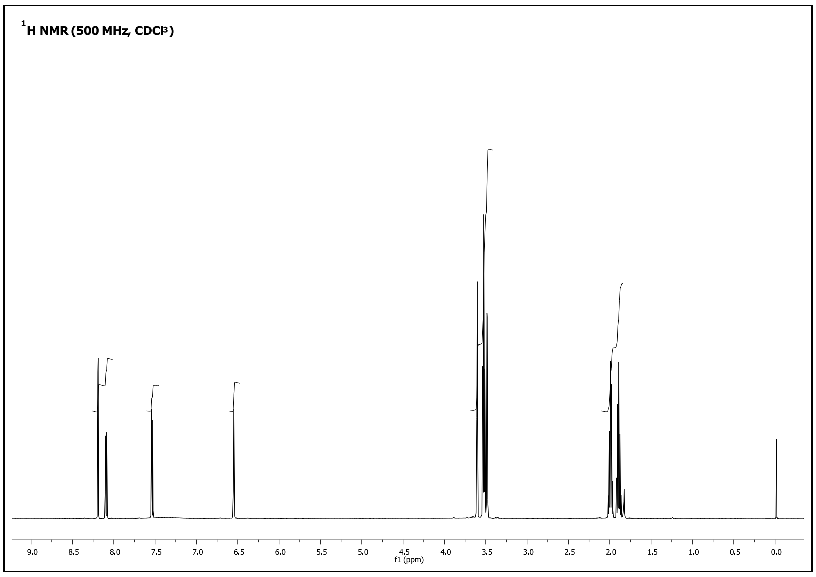

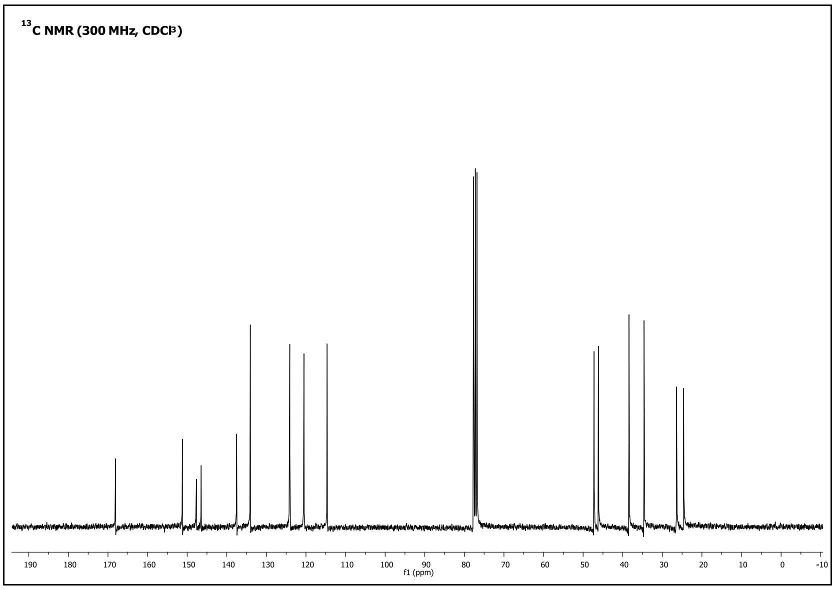

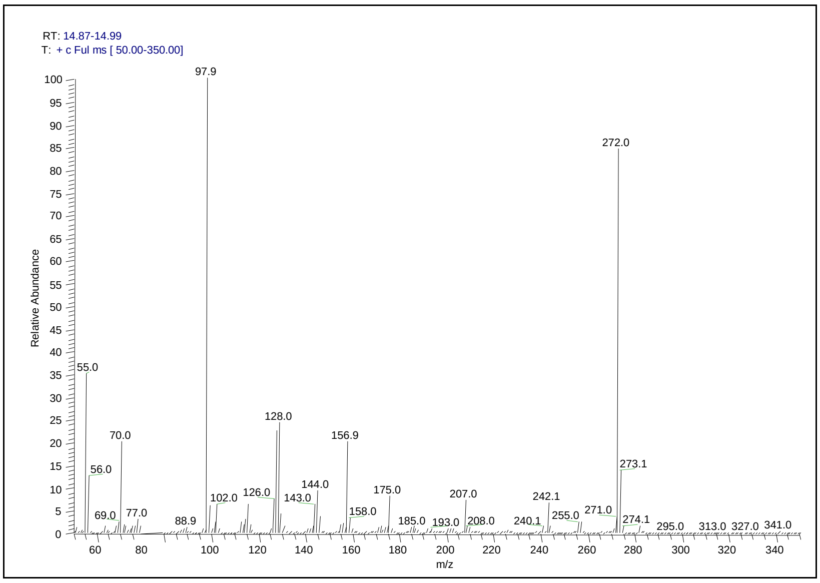

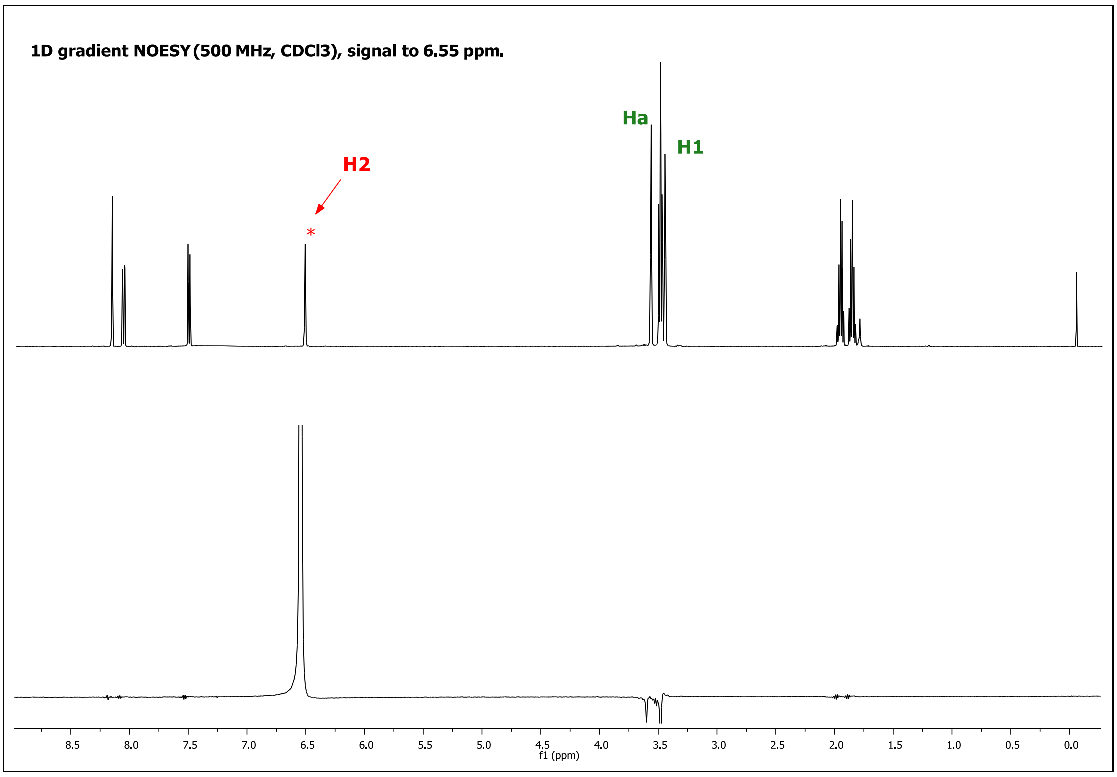
1D gradient NOESY (500 MHz, CDCl3), signal to 6.55 ppm.
Ha
H1
H2
*
8.5
8.0
7.5
7.0
6.5
6.0
5.5
5.0
4.5
4.0
3.5
3.0
2.5
2.0
1.5
1.0
0.5
0.0
f1 (ppm)

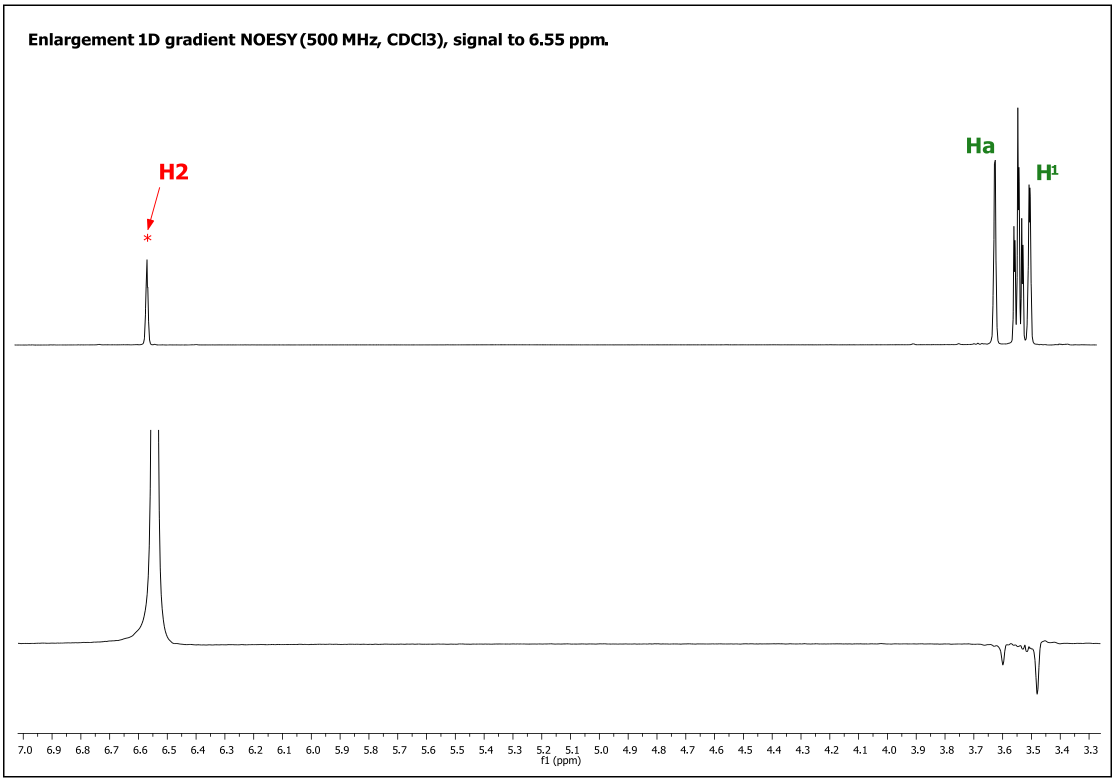
Enlargement 1D gradient NOESY (500 MHz, CDCl3), signal to 6.55 ppm.
Ha
H1
H2
*
7.0 6.9 6.8 6.7 6.6 6.5 6.4 6.3 6.2 6.1 6.0 5.9 5.8 5.7 5.6 5.5 5.4 5.3 5.2 5.1 5.0 4.9 4.8 4.7 4.6 4.5 4.4 4.3 4.2 4.1 4.0 3.9 3.8 3.7 3.6 3.5 3.4 3.3
f1 (ppm)

2-(1,1-Dimethyl-5-nitro-1*H*-inden-3-yl)-*N*,*N*-dimethylacetamide (23)

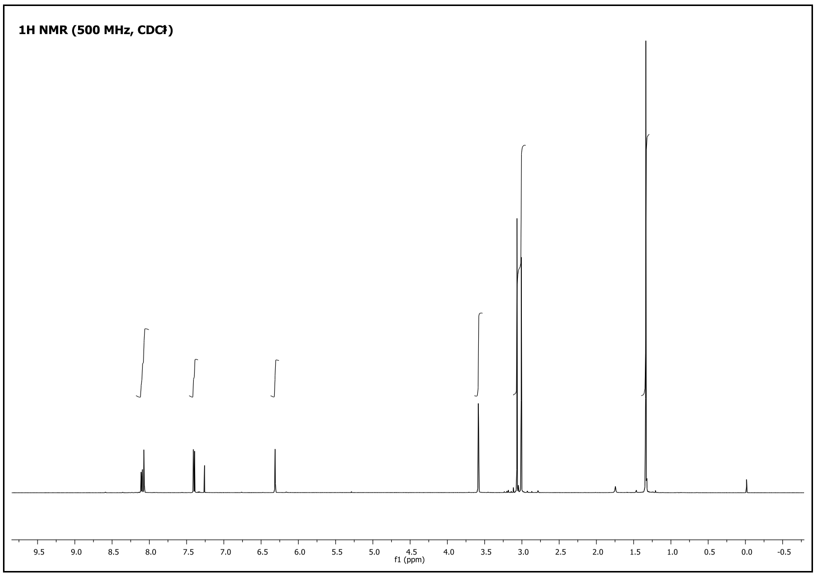

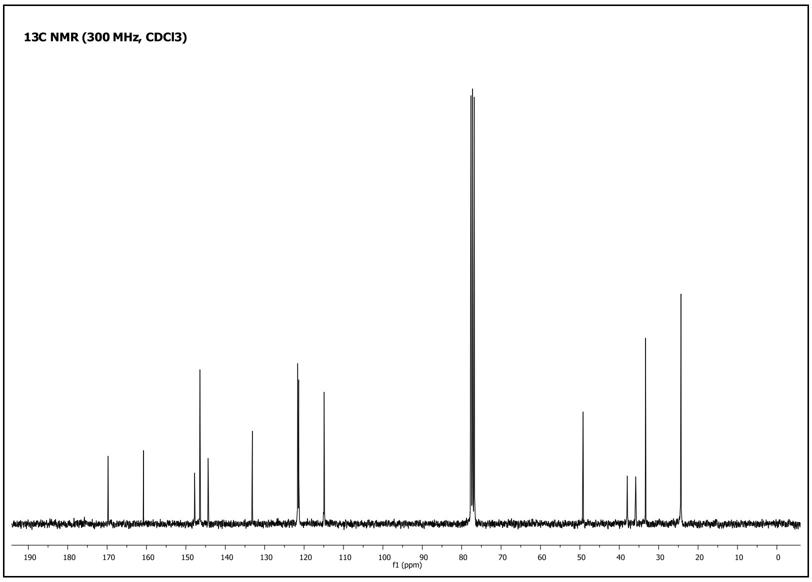

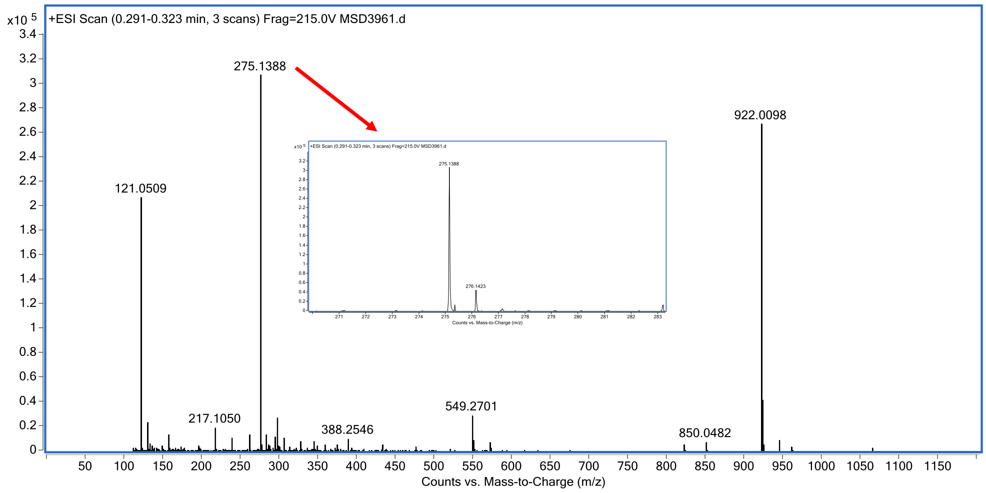

1D gradient NOESY (500 MHz, $CDCl_3$), signal to 6.33 ppm.

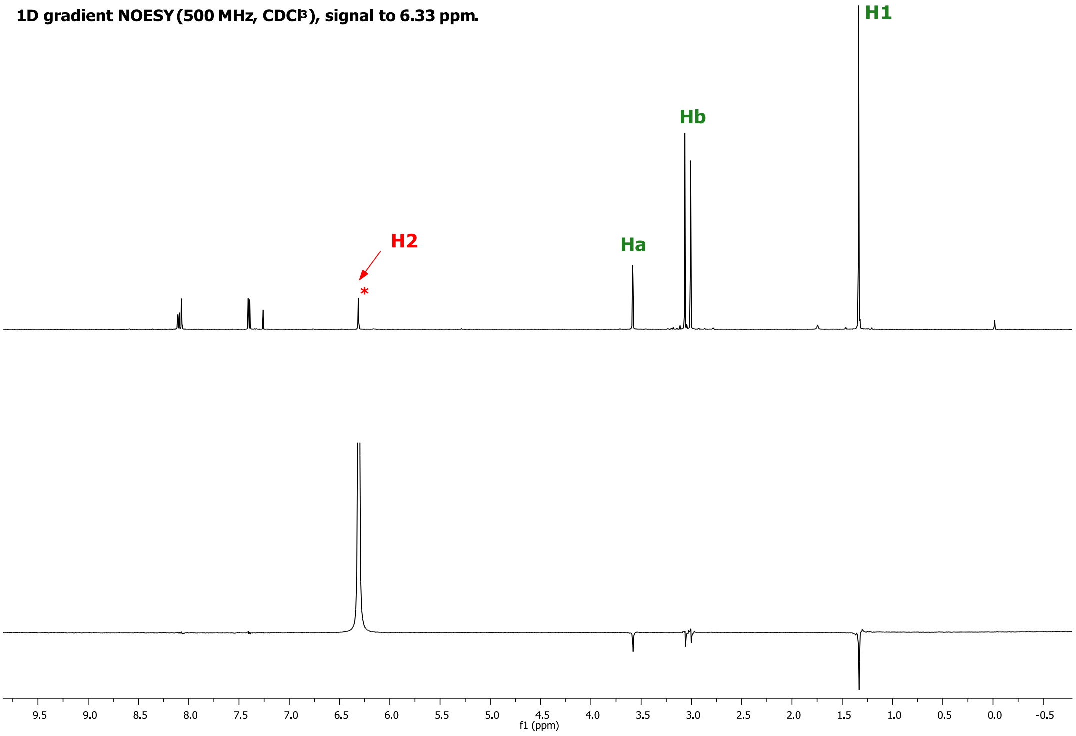

Enlargement 1D gradient NOESY (500 MHz, $CDCl_3$), signal to 6.33 ppm.

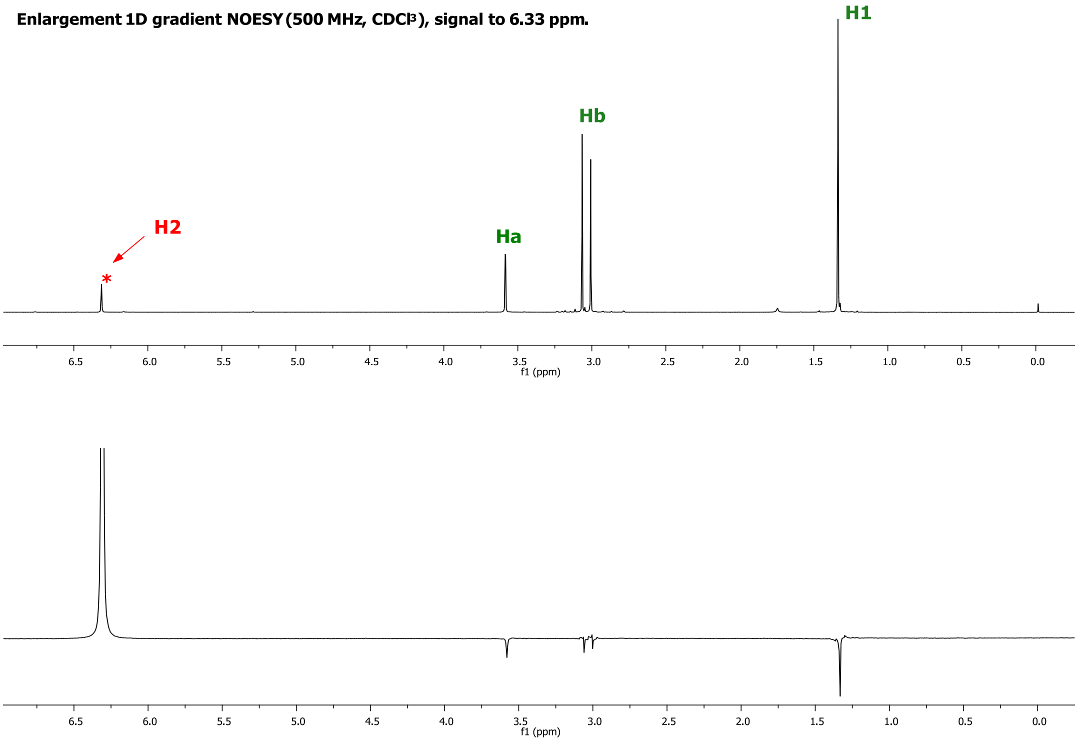

Supplement: File 1 — Assays related to the preparation of 8, 10, 11 and 16, experimental details, characterization data and copies of NMR and ESI-HRMS spectra of all new compounds. [file Beilstein_J_Org_Chem-07-1739-s001.pdf]
